# Editorial: Emerging active, smart and intelligent packaging solutions in the fourth phase of the industrial revolution (Industry 4.0)

**DOI:** 10.3389/fnut.2023.1278143

**Published:** 2023-09-08

**Authors:** Sneh Punia Bungar, Nitya Sharma, Monica Trif, Muhammad Adeel

**Affiliations:** ^1^Department of Food, Nutrition and Packaging Sciences, Clemson University, Clemson, SC, United States; ^2^World Resources Institute India, New Delhi, India; ^3^Department of Food Research, Centre for Innovative Process Engineering (CENTIV) GmbH, Syke, Germany; ^4^BNU-HKUST Laboratory of Green Innovation, Advanced Institute of Natural Sciences, Beijing Normal University at Zhuhai, Zhuhai, Guangdong, China

**Keywords:** active packaging, smart packaging, intelligent packaging (IP), food quality and safety, shelf-life

In the era of Industry 4.0, the fourth phase of the industrial revolution, there has been a remarkable evolution in various sectors, driven by the integration of advanced technologies such as the Internet of Things (IoT), artificial intelligence (AI), big data analytics, and automation (1). One area that has witnessed significant transformation is the packaging industry, where traditional packaging has evolved into a dynamic and responsive system known as Active, Smart, and Intelligent Packaging (ASIP). ASIP can be understood as a collective term for packaging solutions that go beyond the conventional role of containment and protection. These packaging solutions are equipped with embedded technologies that enable them to interact with their environment, monitor product conditions, and provide valuable insights throughout the entire supply chain. This innovation has revolutionized the way products are packaged, delivered, and consumed, leading to improved sustainability, consumer engagement, and supply chain efficiency.

Consumer demands and packaging requirements, including an appetizing and fresh appearance during storage, maximum microbiological safety, no additives, and preservation of nutrients and flavors, are needed in the current entering fourth phase of the industrial revolution (4). The demand for packaged, frozen, and ready-to-eat food has significantly increased in recent years due to changing urban lifestyles and global population patterns. Smart manufacturing processes and systems have received great attention through the latest innovations, ongoing efforts, and best practices. The idea of ASIP is growing very fast, and the global market for smart packaging is expected to reach $26.7 bn by 2024 (2). ASIP is used to extend shelf life, monitor freshness, display quality information, and improve product and customer safety. In addition, smart packaging offers new business opportunities based on digitization and thus fits into the broader realm of Industry 4.0. Further, food waste is a global environmental issue, particularly in industrialized countries that heavily depend on pre-packaged foodstuffs (3). An increase in the use of ASIP packaging can minimize the environmental footprint of packaged food. These packaging systems aim to address food waste by preserving food quality while addressing food safety issues such as the prevention of food-borne diseases and chemical contamination. Moreover, ASIP systems have the potential to promote a circular bio-economy.

This Research Topic presents comprehensive and remarkable reviews and original articles on emerging active, smart, and intelligent packaging solutions trends. A high skepticism regarding nanotechnology foods can be seen compared to nanotechnology food packaging, which is more accepted. ASIP solutions are constantly expanding, and this trend is driving emerging technologies for prolonging shelf life, such as gas scavengers, antioxidants, and antimicrobials. Authors worldwide have submitted manuscripts to this Research Topic (Rzayeva et al.; Ali et al.; Krestyanpol; Chaudhary et al.). Chaudhary et al. provided a valuable perspective on how the distinctive structural and functional characteristics of milk proteins position them as promising contenders for novel active package techniques. These advancements aim to effectively address the requirements of both the food and nutraceutical sectors. Conversely, Ali et al. designed cornstarch-based antimicrobial and edible films using medicinal plants (*Acontium heterophyllum, Artemisia annua*, and *Thymus serpyllum* as fillers. Overall, the articles included in this Research Topic highlight the novel and innovative approach in ASIP.

On behalf of the Guest Editor team, we are pleased to express sincere appreciation for all the contributions to this Research Topic. This Research Topic includes innovative, in-depth research and applications of ASIP in the packaging sector. In addition, we appreciate the invitation and opportunity to edit this Research Topic with the great support of the editorial team of Frontiers in Nutrition.

## Author contributions

SP: Conceptualization, Writing—original draft, Writing—review and editing. NS: Writing—review and editing. MT: Writing—review and editing. MA: Writing—original draft.
